# Curcumin Inhibits Growth of Human NCI-H292 Lung Squamous Cell Carcinoma Cells by Increasing FOXA2 Expression

**DOI:** 10.3389/fphar.2018.00060

**Published:** 2018-02-02

**Authors:** Lingling Tang, Jinjin Liu, Linyun Zhu, Qingge Chen, Ziyu Meng, Li Sun, Junsheng Hu, Zhenhua Ni, Xiongbiao Wang

**Affiliations:** ^1^Department of Respiratory Medicine, Putuo Hospital, Shanghai University of Traditional Chinese Medicine, Shanghai, China; ^2^Central Laboratory, Putuo Hospital, Shanghai University of Traditional Chinese Medicine, Shanghai, China

**Keywords:** lung squamous cell carcinoma, curcumin, FOXA2, proliferation, STAT3

## Abstract

Lung squamous cell carcinoma (LSCC) is a common histological lung cancer subtype, but unlike lung adenocarcinoma, limited therapeutic options are available for treatment. Curcumin, a natural compound, may have anticancer effects in various cancer cells, but how it may be used to treat LSCC has not been well studied. Here, we applied curcumin to a human NCI-H292 LSCC cell line to test anticancer effects and explored underlying potential mechanisms of action. Curcumin treatment inhibited NCI-H292 cell growth and increased FOXA2 expression in a time-dependent manner. FOXA2 expression was decreased in LSCC tissues compared with adjacent normal tissues and knockdown of FOXA2 increased NCI-H292 cells proliferation. Inhibition of cell proliferation by curcumin was attenuated by FOXA2 knockdown. Moreover inhibition of STAT3 pathways by curcumin increased FOXA2 expression in NCI-H292 cells whereas a STAT3 activator (IL-6) significantly inhibited curcumin-induced FOXA2 expression. Also, SOCS1 and SOCS3, negative regulators of STAT3 activity, were upregulated by curcumin treatment. Thus, curcumin inhibited human NCI-H292 cells growth by increasing FOXA2 expression via regulation of STAT3 signaling pathways.

## Introduction

Lung cancer, a highly malignant tumor associated with mortality, can occur as small cell lung cancer (SCLC) or non-small cell lung cancer (NSCLC) (Malanga et al., 2008; Bersaas et al., 2016). Lung squamous cell carcinoma (LSCC), formerly the most common histologic subtype of NSCLC, is characterized by a poor therapeutic response, a high relapse rate, and poor prognosis (Justilien et al., 2014). Unlike lung adenocarcinoma, limited therapeutic options are available for advanced-stage LSCC, therefore, new and better targets or drugs are needed.

Forkhead box transcription factor A2 (FOXA2), or hepatocyte nuclear factor 3 beta (HNF3β), is a member of the forkhead box gene superfamily (Jang et al., 2015). Studies suggest that FOXA2 may be a suppressor of tumor metastasis in human lung cancers (Tang et al., 2011). FOXA2 regulates the expression of genes critical to lung morphogenesis, and loss of FOXA2 expression is frequent in lung cancer cell lines (Khoor et al., 2004). Also, squamous cell carcinomas uniformly lack FOXA2 staining (Khoor et al., 2004). A recent study indicated that long-term tobacco smoke carcinogen exposure downregulated FOXA2 in human bronchial epithelial cells (Bersaas et al., 2016). Therefore, FOXA2 may be an important target protein for therapies against LSCC since LSCCs are linked more strongly with smoking than other forms of NSCLC (Khoor et al., 2004; Basseres et al., 2012), and activation of FOXA2 may be useful for treating human LSCC.

Curcumin is derived from dry rhizomes of the turmeric plant *Curcuma longa*, and it may have activity against aggressive and recurrent cancers, including lung cancer (Mimeault and Batra, 2011; Gupta et al., 2013). Curcumin may inhibit cell proliferation and induce apoptosis (Radhakrishna Pillai et al., 2004; Chen et al., 2010; Jin et al., 2015), enhance chemotherapeutic efficacy (Sen et al., 2005; Chanvorachote et al., 2009), and inhibit metastasis of human lung cancer cells (Liao et al., 2015; Tsai et al., 2015). However, little evidence suggests that curcumin may be useful for LSCC. Zhao et al. (2015) suggested that curcumin reduced cell viability in SK-MES-1 human LSCC cells, and Abbas et al. (2015) reported that curcumin treatment increased endogenous PIAS3 expression and decreased cell growth and viability in Calu-1 human LSCC cells. In NCI-H292 LSCC cells, Haque et al. (2015) and Amin et al. (2015) reported anticancer activity of curcumin and suggested it might target multiple pathways in cells. Thus, we studied whether FOXA2 might participate in anticancer activity of curcumin in NCI-H292 cells.

STAT3 protein is 1 of 7 cytoplasmic transcription factor family members including STAT1-6, STAT5a, and STAT5b (Furqan et al., 2013) which are aberrantly activated in lung cancer tissues (Sanchez-Ceja et al., 2006; Jiang et al., 2016). Moreover, increased STAT3 activity is correlated to poorer overall survival of lung cancer patients (Tong et al., 2017). Thus, STAT3 has been regarded as a key target for lung cancer prevention (Dutta et al., 2014; Harada et al., 2014), and inhibition of STAT3 signal showed great anticancer and antiangiogenic effects *in vitro and in vivo* (Yang et al., 2012; Xu and Zhu, 2017). Recent study showed that activation of STAT3 by *Mycoplasma pneumoniae* inhibited FOXA2 expression in human NCI-H292 LSCC cells (Mishina et al., 2015). It has also been found that curcumin treatment suppressed STAT3 phosphorylation and reduced the proliferative capacity of lung adenocarcinoma-derived H441 cells, indicating STAT3 as a potential target by curcumin in lung cancer (Alexandrow et al., 2012). Perhaps curcumin regulates STAT3-FOXA2 signaling in human NCI-H292 cells.

Here, we describe the anticancer effects of curcumin on cell proliferation in NCI-H292 cells and a potential underlying mechanism that may involve FOXA2.

## Materials and Methods

### Cell Culture

Human NCI-H292 LSCC cell lines were maintained in RPMI medium 1640 (Thermo Fisher Scientific, Waltham, MA, United States) supplemented with 10% fetal bovine serum, penicillin, and streptomycin. Cells were maintained at 37°C in a 5% CO_2_ incubator.

### Tissue Microarray

Tissue microarrays (TMAs) of LSCC were provided by OUTDO Biotech Co., Ltd. (Shanghai, China), containing 89 cases of LSCC cancer tissues and paired adjacent normal tissue. Tissue samples were collected from patients in Taizhou Hospital of Zhejiang Province and approved by the Ethics Committee of Taizhou Hospital of Zhejiang Province. Informed consent was obtained from all patients. Among 89 cases, 81 cases were male, 8 cases were female; 28 cases were under the age of 60 years, and 61 cases were above 60 years in age.

### Immunohistochemistry

In brief, formalin-fixed, paraffin-embedded sections from 89 LSCC patients were deparaffinized and rehydrated. After antigen retrieval using citrate buffer (0.01 mmol/L, pH 6.0), the slides were washed three times with PBS and incubated in 10% normal goat serum to block nonspecific background staining. Sections were then incubated overnight with rabbit anti-human FOXA2 antibodies (Abcam, Cambridge, United Kingdom) at 4°C. After incubation, tissue sections were washed with PBS and treated with a streptavidin-biotin-peroxidase complex (SABC kit, Boster, Wuhan, China). Signal detection was performed using diaminobenzidine (DAB). Immunohistochemical staining was assessed semi-quantitatively by measuring intensity of the staining (0, 1, 2, or 3) and extent of staining (0, 0%; 1, 0–10%; 2, 10–50%; 3, 50–100%). Intensity scores and extent of staining were multiplied to give a weighted score for each case. For statistical analysis, weighted scores were grouped in two categories for which scores of 0–3 were considered negative and 4–9 were positive.

### Cell Viability Assays

Cell viability was measured by using a Cell Counting Kit-8 (CCK-8, Dojindo, Japan) according to the manufacturer’s instructions. Briefly, cells were seeded at a density of 0.5 × 10^4^ cells/well in 96-well plates, and incubated in RPMI medium 1640 supplemented without FBS overnight. Curcumin (Selleck, Houston, United States) was dissolved in dimethyl sulfoxide (DMSO). Cells were treated with various concentrations (0, 5, 10, 20, and 40 μM) of curcumin for 24 h, and appropriate controls were treated with DMSO at the same concentrations. The samples were tested every 24 h for 3 days. CCK-8 solution (10 μl) was added to each well for 2 h and optical density was measured at 450 nm to estimate viable cells.

### Apoptosis Analysis

An Annexin V, 633 Apoptosis Detection Kit (Dojindo, Japan) was used according to the manufacturer’s protocol. In brief, harvested cells were mixed, washed twice with PBS and resuspended in binding buffer at a final density of 10^6^ cells/ml. Annexin V-633 (5 μl) were added to 100 μl of the cell suspension containing 10^5^ cells. The cell suspension was mixed and then incubated for 15 min at room temperature in the dark. Subsequently, 200 μl of binding buffer were added and cells were analyzed by flow cytometry using Calibur (BD Bioscience, CA, United States).

### Caspase-3/7 Activity Analysis

Caspase-3/7 activity was carried out using the Caspase-Glo 3/7 assay kit (Promega, Madison, WI, United States) according to the manufacturer’s protocol. Briefly, NCI-H292 cells treated by curcumin with different dosages (10 and 40 μM). After 24 h treatment, Caspase-Glo 3/7 reagent was added to 96-well plates. Plates were gently shaken and then incubated in the dark at 37°C for 2 h and Caspase-3/7 activity was recorded using GloMax 20/20 Luminometer (Promega, Shanghai, China).

### Gene Silencing

Small-interfering RNAs targeting human FOXA2 (Gene ID: 207) were synthesized (GenePharma, Shanghai, China) and transfected into cells with TransIT-TKO (Mirus, Madison, WI, United States). Scrambled siRNA was a control. Sequences of siRNA used for FOXA2 silencing were as follows: FOXA2 siRNA, 5′-ACCAGTGGATCATGGACCT-3′; control siRNA, 5′-TTCTCCGAACGTGTCATGT-3′.

### Real-Time Quantitative PCR (qRT-PCR)

Total RNA was isolated from NCI-H292 cells using Trizol reagent (TaKaRa, Dalian, China), and first-strand cDNAs were prepared using a random hexamer primer according to the instructions included with the First-Strand Synthesis Kit (Roche, San Francisco, CA, United States). PCR were performed using specific forward and reverse primers (FOXA2, forward, 5′-GGAGCAGCTACTATGCAGAGC-3′, reverse, 5′-CGTGTTCATGCCGTTCATCC-3′; β-actin, forward, 5′-CCAACCGCGAGAAGATGA-3′, reverse, 5′-CCAGAGGCGTACAGGGATAG-3′). Real-time PCR was performed using Universal Master Mixer (Roche, Switzerland). Relative expression of the FOXA2 gene was normalized against β-actin and analyzed via the 2-ΔΔCt method.

(1) $$[\mathrm{\Delta \Delta Ct}=({\mathrm{Ct}}_{\mathrm{Target}}-{\mathrm{Ct}}_{\mathrm{Reference}})\mathrm{sample}-({\mathrm{Ct}}_{\mathrm{Target}}-{\mathrm{Ct}}_{\mathrm{Reference}})\mathrm{control}].$$

### Western Blot Analysis

Western blot analysis was performed according to published methods (Ni et al., 2016). Cells were washed once PBS and dissolved in cell lysis reagent (Cell Signaling Technology, Danvers, MA, United States). Total protein was separated with 10% SDS–PAGE, followed by transfer to polyvinylidene difluoride (PVDF) membrane. The PVDF membrane was blocked with 5% BSA, washed three times with TBST, and then incubated with the antibodies separately overnight at 4°C. Antibodies to β-actin, P-STAT3, and P-STAT6 were from Cell Signaling (Cell Signaling Technology, Danvers, MA, United States) and antibodies to FOXA2 were from Santa Cruz (Santa Cruz Biotechnology, Dallas, TX, United States). The membrane was then washed with TBST three times followed by incubation with anti-rabbit IgG or anti-mouse IgG horseradish peroxidase secondary antibody (Cell Signaling Technology, Danvers, MA, United States) for 2 h at room temperature. Finally, immunoreactive bands were visualized with ECL reagent. Relative protein expression levels were quantified by using Image J software and normalized to β-actin.

### Nuclear Protein Extraction

The NCI-H292 cells were harvested and nuclear protein fractions were isolated using a NE-PER Nuclear and Cytoplasmic Extraction kit (Thermo Fisher Scientific, Waltham, MA, United States) according to the manufacturer’s instructions.

### Statistical Analysis

The SPSS version 21.0 software was used for data analyses. Statistical significance was confirmed using a Student’s *t*-test or a Mann–Whitney *U*-test. Statistically significant differences were defined as *p* < 0.05.

## Results

### Effects of Curcumin on Proliferation and Apoptosis in NCI-H292 Cells

**FIGURE 1:**
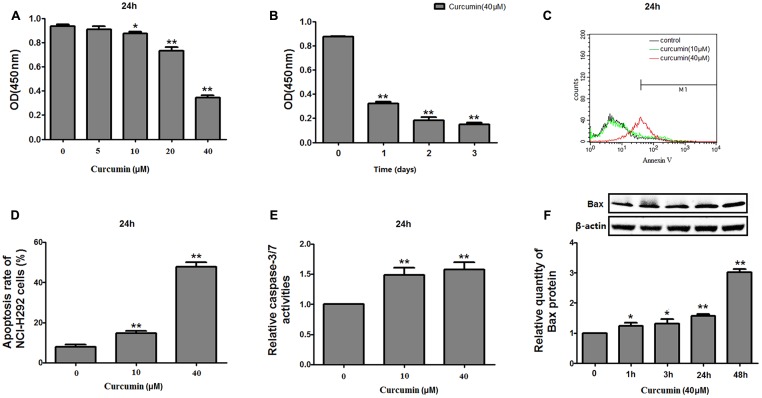
Effects of curcumin on proliferation and apoptosis of NCI-H292 cells. NCI-H292 cells were treated with curcumin at the doses indicated or vehicle (DMSO, control) at different time intervals, following which cell viability (CCK-8) assays **(A,B)** and cell apoptosis assays **(C,D)** were performed to determine growth suppressive effects of curcumin. Cell lysates after curcumin treatment were subjected to Caspase-Glo 3/7 activity analysis **(E)** and protein expression analysis with antibodies indicated **(F)**. Data are presented as mean ± SEM from three independent, ^∗^*p* < 0.05, ^∗∗^*p* < 0.01.

The effect of curcumin on NCI-H292 proliferation was reconfirmed using a CCK-8 method. Curcumin showed a dose- and time-dependent inhibition on the growth of NCI-H292 cells (**Figures 1A,B**), likely due to increased apoptosis. An Annexin V apoptosis assay revealed that curcumin promoted NCI-H292 cells apoptosis in a dose-dependent manner (**Figures 1C,D**). This dose-dependent apoptotic response was measured by a luminescent-based Caspase-3/7 assay. The results showed increased Caspase-3/7 activity with curcumin treatment (**Figure 1E**). In addition, pro-apoptotic Bax protein was upregulated by curcumin (**Figure 1F**). Thus, curcumin treatment caused apoptosis and reduced proliferation of NCI-H292 cells.

### FOXA2 Was Responsible for Curcumin-Mediated Cell Growth Inhibition

**FIGURE 2:**
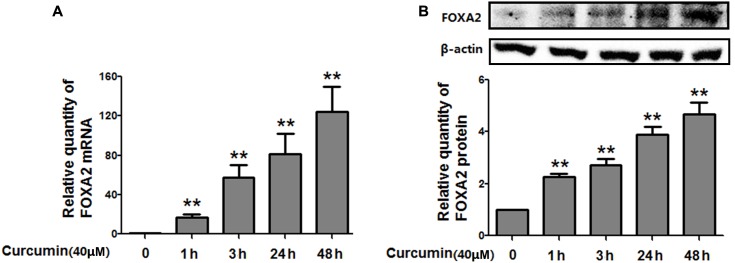
Curcumin increased the expression of FOXA2 in NCI-H292 cells. Cultured NCI-H292 cells were treated with curcumin (40 μM) or vehicle (DMSO, control) at different time intervals. The mRNA and protein expression level of FOXA2 were determined via qRT-PCR **(A)** and western blotting **(B)**, respectively. Data are presented as mean ± SEM from three independent, ^∗^*p* < 0.05, ^∗∗^*p* < 0.01.

**FIGURE 3:**
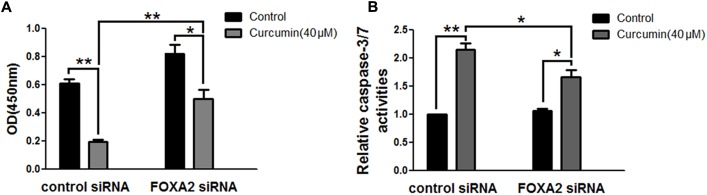
Curcumin inhibited NCI-H292 growth in FOXA2 dependent manner. The siRNA duplexes against FOXA2 were transfected into NCI-H292 cells, and then incubated in the presence of curcumin (40 μM) or vehicle (DMSO, control) for 24 h, following which cell viability (CCK-8) assays **(A)** and Caspase-Glo 3/7 activity assays **(B)** were performed to determine growth suppressive effects of curcumin. Data are presented as mean ± SEM from three independent, ^∗^*p* < 0.05, ^∗∗^*p* < 0.01.

To investigate whether FOXA2 was involved in the curcumin-mediated inhibition of cell growth of NCI-H292 cells, we examined the expression level of FOXA2 after curcumin treatment. As shown in **Figures 2A,B**, curcumin treatment markedly increase FOXA2 mRNA and protein expression in a time-dependent manner. We then studied whether curcumin inhibited NCI-H292 growth in a FOXA2-dependent manner using FOXA2 siRNA. Curcumin-induced cell proliferation inhibition was rescued after FOXA2 knockdown compared to controls (**Figure 3A**). The inhibitory rate of curcumin was significantly decreased in FOXA2 knockdown cells compared to controls (inhibitory rate: 40% vs. 60%). Also, Caspase-3/7 activation was attenuated (**Figure 3B**), suggesting that curcumin inhibited NCI-H292 cells growth in FOXA2-dependent manner.

### FOXA2 Expression Was Frequently Lost in LSCC Tissues and Associated with Cell Proliferation

**FIGURE 4:**
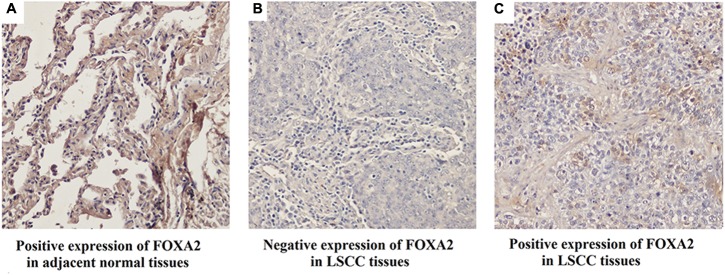
Expression of FOXA2 in human LSCC tissues and adjacent normal tissues. **(A)** Positive expression of FOXA2 in adjacent normal tissues. **(B)** Negative expression of FOXA2 in LSCC tissues. **(C)** Positive expression of FOXA2 in LSCC tissues. ^∗∗^*p* < 0.01 vs. adjacent tissues.

**FIGURE 5:**
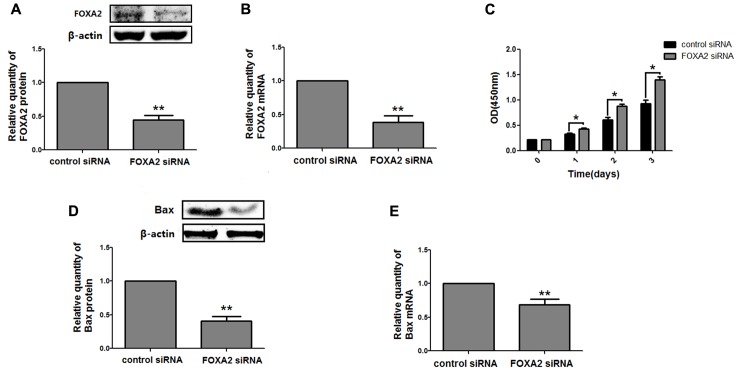
Knockdown of FOXA2 increased cell proliferation. The siRNA duplexes against FOXA2 were transfected into NCI-H292 cells, and the mRNA and protein expression level of FOXA2 and Bax were analyzed using western blotting **(A,D)** and qRT-PCR **(B,E)** at 24 h after the siRNA transfections. **(C)** FOXA2 silenced NCI-H292 cells were incubated in the 96-well plates for 1, 2, and 3 days, following which CCK-8 assays were performed to determine cell viability. Data are presented as mean ± SEM from three independent, ^∗^*p* < 0.05, ^∗∗^*p* < 0.01.

Next, we measured the expression of FOXA2 in LSCC tissues. Data showed that FOXA2 was downregulated in LSCC tissues compared with adjacent normal tissues (**Figure 4**). Weak expression or loss of expression of FOXA2 occurred in more than half of samples analyzed (**Table 1**). These results suggested that loss of FOXA2 expression might play important roles in the tumourigenesis of LSCC. The role of FOXA2 on cell proliferation was then assessed in NCI-H292 cells using RNA interference. **Figures 5A,B** showed that FOXA2 mRNA and protein were reduced by FOXA2 siRNA treatment. The growth of NCI-H292 cells transfected with FOXA2 siRNA was significantly increased after 1, 2, and 3 days compared with the control cells (**Figure 5C**). Recent study identified Bax as a putative target gene of FOXA2 in lung adenocarcinoma cells by screening techniques (Jang et al., 2015). Also, we found pro-apoptotic Bax protein and mRNA decreased after FOXA2 knockdown in NCI-H292 cells (**Figures 5D,E**). Our results demonstrated that FOXA2 expression was frequently lost in LSCC tissues and loss of FOXA2 expression increased proliferation of NCI-H292 cells.

**Table 1:** FOXA2 expression in human LSCC tissues.

Tissue	Case	FOXA2 expression		*p*-value
		Negative	Positive	
Tumors	89	59	30	0.000
Adjacent tissues	89	16	73	–

### Inhibition of STAT3 Pathway by Curcumin Increased FOXA2 Expression in NCI-H292 Cells

**FIGURE 6:**
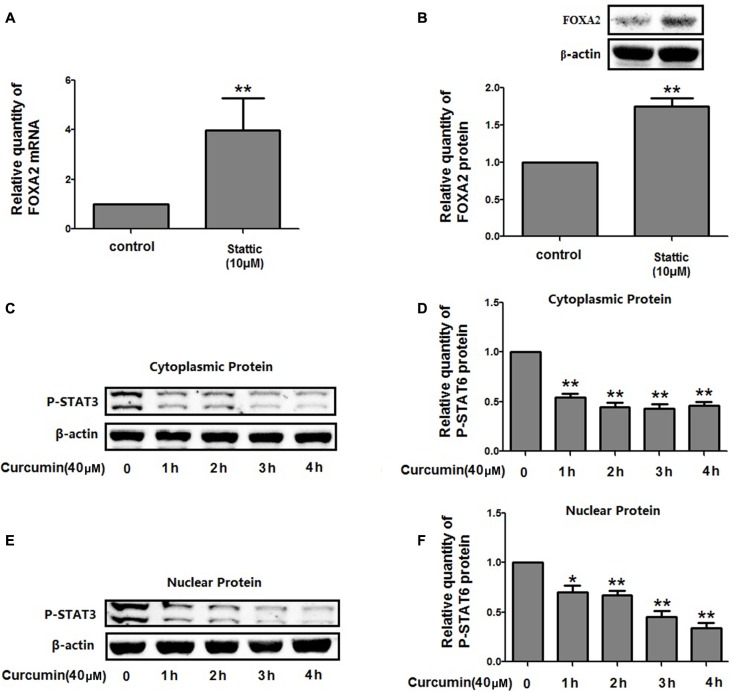
Curcumin inhibited STAT3 pathway in NCI-H292 cells. **(A,B)** Cultured NCI-H292 were treated with stattic (10 μM) or vehicle (DMSO, control) for 24 h, the mRNA and protein expression level of FOXA2 were analyzed using qRT-PCR and western blotting. **(C–F)** Cultured NCI-H292 were treated with curcumin (40 μM) or vehicle (DMSO, control) for 0–4 h. P-STAT3 level in the cytoplasmic and nuclear fraction was detected by western blotting. Data are presented as mean ± SEM from three independent, ^∗^*p* < 0.05, ^∗∗^*p* < 0.01.

**FIGURE 7:**
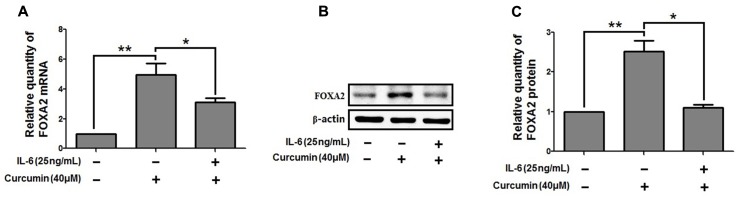
STAT3 pathway was responsible for the increased effect of curcumin on FOXA2 expression. NCI-H292 cells were treated with IL-6 (25 ng/mL) plus curcumin (40 μM) or vehicle (DMSO, PBS-0.1% BSA, control) for 24 h. The mRNA and protein expression level of FOXA2 were analyzed by using qRT-PCR **(A)** and western blotting **(B,C)**. Data are presented as mean ± SEM from three independent, ^∗^*p* < 0.05, ^∗∗^*p* < 0.01.

We then investigate the molecular mechanisms underlying FOXA2 regulation by curcumin. It was reported that the STAT3 pathways might be the upstream regulator of FOXA2 (Mishina et al., 2015), and curcumin was a STAT3 inhibitor (Alexandrow et al., 2012). We found that treatment with a STAT3 inhibitor (stattic) increased FOXA2 expression similarly in NCI-H292 cells (**Figures 6A,B**). We then measured phosphorylation of STAT3 in NCI-H292 cells treated with curcumin. P-STAT3 cytoplasmic protein was reduced by curcumin treatment as was nuclear protein downregulation (**Figures 6C–F**). Our results confirmed that curcumin could inhibit STAT3 pathways in NCI-H292 cells, which was consistent with previous study (Alexandrow et al., 2012). We next investigated whether curcumin increased FOXA2 in STAT3-dependent manner. After treatment with STAT3 activator (IL-6) plus curcumin, expression of FOXA2 was reduced compared with untreated activator-stimulated NCI-H292 cells (**Figure 7**). Thus, curcumin increased FOXA2 expression by inhibiting STAT3 signaling.

### Curcumin Increased the Expression Level of SOCS1 and SOCS3 in NCI-H292 Cells

**FIGURE 8:**
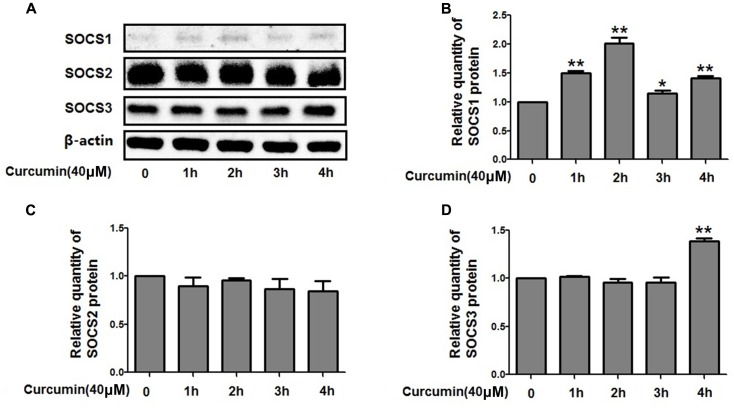
Effects of curcumin on SOCSs expression in NCI-H292 cells. **(A–D)** Cultured NCI-H292 were treated with curcumin (40 μM) or vehicle (DMSO, control) for 0–4 h. Cell lysates were subjected to protein expression analysis with antibodies indicated. Data are presented as mean ± SEM from three independent, ^∗^*p* < 0.05, ^∗∗^*p* < 0.01.

The expression of suppressors of cytokine signaling proteins (SOCS1, SOCS2, and SOCS3), the negative regulators of STAT3, was examined by western blot analyses. The relative protein levels of SOCS2 were unchanged but increased for SOCS1 and SOCS3 after curcumin treatment (**Figure 8**). Therefore, it is likely that curcumin increased the expression level of SOCS1 and SOCS3, thus inhibiting the activity of STAT3 which resulted in change of FOXA2 expression.

## Discussion

LSCC is a NSCLC representing 30% of all lung cancer cases (Justilien et al., 2014). Unlike lung adenocarcinoma, activating mutations in EGFR and ALK fusion are typically not present in LSCC, so targeted agents are largely ineffective against LSCC (Cancer Genome Atlas Research Network, 2012). Our data agree with previous studies that curcumin inhibited cell proliferation of LSCC NCI-H292 cells in a time- and dose-dependent manner (Amin et al., 2015; Haque et al., 2015). Thus, curcumin may be an adjuvant for LSCC therapy. Also, expression of FOXA2 was decreased in LSCC patients and curcumin’s ability to slow NCI-H292 cells growth might be associated with increasing FOXA2 expression, suggesting that FOXA2 is a novel target of curcumin.

How FOXA2 participates in lung cancer pathogenesis is not very clear. In NCI-H358 human lung adenocarcinoma cells, Halmos’ group reported that overexpression of FOXA2 reduced growth, arrested proliferation, and increased apoptosis (Halmos et al., 2004). We noted that growth of NCI-H292 cells transfected with FOXA2 siRNA significantly increased compared with controls, suggesting that FOXA2 may be an important regulator for squamous cell carcinoma cell and adenocarcinoma cell proliferation. Jang et al. (2015) screened the FOXA2-mediated transcriptional regulation network in NSCLC and identified Bax as a putative target gene of FOXA2. We confirmed that Bax was regulated by FOXA2, which decreased after FOXA2 knockdown. Also, curcumin treatment increased Bax expression in NCI-H292 cells; therefore, cancer cell growth inhibition by curcumin may be due to FOXA2-induced Bax upregulation.

To investigate the molecular mechanisms underlying FOXA2 regulation by curcumin, phosphorylation of STAT3 in NCI-H292 cells was measured. We found curcumin-mediated FOXA2 upregulation was associated with inhibition of STAT3 signals. STAT3 proteins are expected to promote gene transcription by binding to specific DNA sites as homo- or heterodimers in the promoter region (Mascareno et al., 1998). However, studies suggest that STAT3 transcription factors can act as transcriptional repressors by interfering with other transcription factors (de la Iglesia et al., 2008; Bedel et al., 2011). Meanwhile other studies indicated that transcription factor SPDEF was an upstream regulator for FOXA2 expression (Yu et al., 2010; Du et al., 2017). We found no significantly different changes in the expression of SPDEF after curcumin treatment of NCI-H292 cells (data not shown), suggesting SPDEF might not be involved in curcumin-mediated FOXA2 expression. Therefore, more work is required to validate our preliminary findings whether STAT3 inhibited FOXA2 expression by direct binding to the putative site in the FOXA2 promoter region, or by interfering with other transcription factors.

Recent studies indicate that curcumin’s pleiotropic anti-tumoral activity may be related to the modulation of numerous signaling molecules such as NF-κB, AP-1, JAK/STAT, MAPK, Nrf-2, AKT, and PPAR (Gupta et al., 2013; Shehzad and Lee, 2013; He et al., 2016). Yang et al. (2012) investigated protein changes upstream of STAT3 in SCLC cells and found that inhibition of STAT3 phosphorylation was due to inhibition of JAK because JAK phosphorylation was intermediately suppressed by curcumin treatment. In our studies, curcumin also decreased the phosphorylation of STAT3 in both the nucleus and the cytoplasm in NCI-H292 cells. To better understand the mechanisms underlying curcumin-mediated suppression of STAT3 activation, we analyzed proteins upstream of STAT-3. Our data showed SOCS1 and SOCS3 was upregulated by curcumin treatment. SOCSs are the cytokine inducible endogenous inhibitors of STAT-3, which bind through the SH2 domain to phosphotyrosine residues in either cytokine receptors or JAK and suppress STAT-3 signaling (Saydmohammed et al., 2010). We found curcumin treatment increased SOCS3 expression around 4 h post-stimulation and increased SOCS1 protein appeared earlier (1 or 2 h) and this was accompanied by a decrease of phosphorylated STAT3, suggesting that enhancement of SOCS1 protein was likely to be one of the mechanisms contributing to the early inhibition of STAT3 signal in NCI-H292 cells. Chen et al. (2013) indicated a regulatory mechanism of SOCS1 and SOCS3 through inhibition of HDAC activity (especially HDAC8) by curcumin in myeloproliferative neoplasms. Whether a similar mechanism for curcumin exists in NCI-H292 cells is not clear.

In summary, we tested and reported the anticancer effects of curcumin in human NCI-H292 LSCC cells and explored the potential mechanistic roles. Our results suggested the role of STAT3 in the upregulation of FOXA2 expression, which was targeted by curcumin. And the induction of FOXA2 by curcumin was one of the mechanisms through which curcumin inhibited NCI-H292 cells growth. In addition, our studies showed curcumin increased the expression level of SOCS1 and SOCS3, the negative regulators of STAT3. SOCS1 and SOCS3 might thus be part of novel targets for curcumin to mediate STAT3-FOXA2 signaling in NCI-H292 cells.

## Author Contributions

ZN and XW conceived and designed the experiments. LT, JL, LZ, QC, and ZM performed the experiments. LT, LS, and JH analyzed the data. LT, ZN, and XW wrote the manuscript. XW contributed to English editing. All authors reviewed the results and approved the final version of the manuscript.

## Conflict of Interest Statement

The authors declare that the research was conducted in the absence of any commercial or financial relationships that could be construed as a potential conflict of interest.
